# Hydroalcoholic extract of *Haematoxylum brasiletto* protects *Caenorhabditis elegans* from cadmium-induced toxicity

**DOI:** 10.1186/s12906-022-03654-6

**Published:** 2022-07-11

**Authors:** Margareth Duran-Izquierdo, María Taboada-Alquerque, Lucellys Sierra-Marquez, Neda Alvarez-Ortega, Elena Stashenko, Jesus Olivero-Verbel

**Affiliations:** 1 Environmental and Computational Chemistry Group, School of Pharmaceutical Sciences, Zaragocilla Campus, University of Cartagena, 130014 Cartagena, Colombia; 2 Center for Chromatography and Mass Spectrometry, CROM-MASS, CIBIMOL-CENIVAM, Industrial University of Santander, Carrera 27, Calle 9, Building 45, 680002 Bucaramanga, Colombia

**Keywords:** Nematode, Oxidative stress, Fabaceae, Daf-16, Biodiversity

## Abstract

**Background:**

*H. brasiletto* is used in popular culture due to its therapeutic properties, including antioxidant, anti-inflammatory and antiproliferative properties, although little is known about its role as a protector against metal toxicity. This study aimed to investigate the chemical composition and efficacy of the hydroalcoholic extract from *H. brasiletto* (HAE-*Hbrasiletto*) collected in northern Colombia to defend against cadmium (Cd)-induced toxicity.

**Methods:**

Phytochemical characterization was performed using HPLC-ESI-QTOF. *Caenorhabditis elegans* was employed to assess the shielding effect of HAE-*Hbrasiletto* against Cd toxicity in vivo, and the 2,2-diphenyl-1-picrylhydrazyl (DPPH) assay was utilized to measure radical scavenging activity.

**Results:**

The main secondary metabolites identified by HPLC-ESI-QTOF in the extracts were hematoxylins (brazilein and hematein) and protosappanins (protosappanin A, B and C, 10-O-methylprotosappanin B, and protosappanin A dimethyl acetal). The HAE-*Hbrasiletto* elicited low lethality in N2 worms and significantly reduced the Cd-induced death of the nematodes. It also improved Cd-induced motility inhibition, as well as body length and reproduction reduction provoked by the heavy metal. The extract displayed a good capacity to halt Cd-induced DAF-16 translocation. As this last process was associated with lethality (r = 0.962, *p* < 0.01), the antioxidant properties of the extract may contribute to ameliorating tissue damage induced by oxidative stress from Cd exposure.

**Conclusion:**

HAE-*Hbrasiletto* has remarkable properties to protect against Cd-induced toxicity.

**Supplementary Information:**

The online version contains supplementary material available at 10.1186/s12906-022-03654-6.

## Highlights

- Most secondary metabolites tentatively identified in *H. brasiletto* are homoisoflavones.
- Hidroalcoholic extract of *H. brasiletto* protects *C. elegans* from Cd toxicity
- The extract diminished Cd-induced damage to reproduction, growth, and locomotion.
- Cd-induced oxidative stress and translocation of DAF-16 are blocked by the extract.

**Supplementary Information:**

The online version contains supplementary material available at 10.1186/s12906-022-03654-6.

## Background

Plants are an important source of new chemicals that could be used therapeutically [1, 2]. They have been used for years as alternatives to traditional medicine, especially in rural communities lacking access to health services (World Health Organization (WHO) [3]. In plants, secondary metabolites possess biological and pharmacological activities [4]. However, only approximately 15% of the plant species have been explored for studies of biologically active molecules [5]. Colombia is on the list of countries with the greatest biodiversity and is considered the second in the global scenario in terms of plants (30,736 species) [6]. Moreover, the presence of diverse biomes provides excellent habitats for the growth of species with different characteristics, an essential feature in the development of therapies based on ethnopharmacology [7].

The promising biological activities of plants, their extracts, or isolated compounds have been evaluated using in vitro and in vivo studies, and although the number of available assays is immense, the use of the nematode *Caenorhabditis elegans* and its transgenic strains has increased in recent years as a discovery tool in several areas of pharmacological research dealing with natural products [8, 9], especially for screening extracts and isolated bioactive compounds [10]. This versatility of *C. elegans* arises from multiple arrays of sublethal endpoints that can be evaluated [11], together with a large number of mutant strains that allow the study of transcriptional monitoring in vivo [12].

The nematode can also be utilized to assess the effect of natural products in animal models of chemical-induced toxicity [13], although few have involved heavy metals, despite the recognized effects of these elements on *C. elegans*. For instance, *C. elegans* exposed to cadmium (Cd), a well-known inducer of oxidative stress [14], displays good concentration–response relationships for sublethal endpoints [15]. It is also known that nematodes exposed to oxidative stress undergo nuclear translocation of DAF-16 protein [16], the *C. elegans* homolog of the forkhead box transcription factor class O (FoxO), making this process a suitable endpoint for monitoring increased levels of intracellular reactive oxygen species (ROS) [17].

Among the vast number of plants with extracts that could be used to counteract the toxic effects of pollutants, *Haematoxylum brasiletto* H. Karst (Fabaceae), native to Central America, commonly known as “palo de Brasil, palo de tinta, palo tinto or Brasil”, is used in different countries by rural and indigenous communities as a dyeing agent and for medicinal purposes related to diabetes, kidney problems, hypertension and stomach upsets [18, 19], diseases whose symptoms have also been linked to metal exposure [20]. In vitro studies have documented several pharmacological properties of *H. brasiletto*, including antiproliferative, antibacterial [21, 22], and anti-*Trypanosoma cruzi* activities [23]. In Colombia, *H. brasiletto*, known as “barasin”, is found in the tropical dry forest and is mainly used by indigenous communities as a pigment. Despite these properties, there is little information regarding its use as a phytotherapeutic agent to control toxicity induced by heavy metals, especially because several of the effects promoted by these chemicals, such as oxidative stress and cell proliferation [24], have been reported to be ameliorated by *H. brasiletto* [19]. The aims of this study were to characterize the different chemical components present in the extract from a Colombian sample, to investigate the potential of a hydroalcoholic extract of *H. brasiletto* to protect *C. elegans* from Cd-induced toxicity and to evaluate its antioxidant activity.

## Methods

### Reagents

Cadmium chloride (99.99% trace metals basis) was obtained from Sigma-Aldrich (St. Louis, MO 63,103 USA). A Cd solution was prepared using Milli-Q water, and subsequent dilutions were made with K-medium (52 mM NaCl and 32 mM KCl in Milli-Q water). Acetonitrile (ACN) and water (all of LC/MS grade) were purchased from Merck (Darmstadt, Germany), and formic acid (98%) was obtained from Panreac AppliChem (Darmstadt, Germany).

### Plant material and extraction

The plant material (bark) of *H. brasiletto* was collected by Naguib Peñates-Pereira and Wilmer Peñates-Hernández in Carmen de Bolivar (9°43′33.46’’N, 75°5′12.21’’W), northern Colombia. Plant collection permit was obtained from the Ministerio de Ambiente y Desarrollo Sostenible (Colombia) under Resolution 1761 of November 1, 2019. Specimen identification was carried out by biologist Angee Gomez-Gamarra, and the corresponding voucher was deposited at the Universidad de Sucre Herbarium (Colombia) under Herbarium Code 5260. The bark was quickly washed with Milli-Q cold water, finely cut and dried under vacuum. The dried plant material was ground, and 200 g was subjected to extraction for 48 h with a mixture containing ethanol (99.9%) and Milli-Q water in a 70:30 ratio (Vol/Vol). After 24 h, the extract was filtrated with Whatman paper, and the plant material was again subjected to hydroalcoholic extraction for another 24 h. The combined extracts were rotary evaporated up to 40 mL and then freeze dried. The yield of the extract was calculated to be 7.5%. Samples were stored at -20 °C until used. All methods were performed in accordance with the relevant guidelines and regulations, both at the national and international levels.

### Analysis of the HAE-*Hbrasiletto* by HPLC-QTOF-MS/MS

The analysis of chemical constituents tentatively identified in the hydroalcoholic extract from *H. brasiletto* (HAE-*Hbrasiletto*) was conducted using HPLC-QTOF-MS/MS, following recommendations from the literature. The method is described in the Supplementary Material.

### *Caenorhabditis elegans* strains, maintenance, and synchronization

Wild-type N2 (var. Bristol) and transgenic TJ356 worms (zIs356 [daf-16p::daf-16a/b::GFP + rol-6]) were used in this study. The worms and the *Escherichia coli* OP50 strain were obtained from the Caenorhabditis Genetics Center (CGC) (University of Minnesota, MN, USA). *C. elegans* was maintained at 20 °C in Petri dishes on nematode growth medium [NGM] (KCl, NaCl, agar, peptone, cholesterol, CaCl_2_ and MgSO_4_; pH 7.0) and seeded with *E. coli* OP50 as a standard food source [25] For maintenance, all nematode stages were transferred to new plates on a weekly basis and regularly monitored. All experiments were performed using synchronized worms. To this end, adult stage gravid hermaphrodites were lysed using 10 N NaOH and 2.5% NaClO. Isolated eggs were pelleted and washed with K-medium. Toxicity endpoints such as lethality, growth, and locomotion behaviors were assessed employing the N2 strain, whereas the transgenic strain was utilized to examine DAF-16 translocation.

### Experimental design

For biological assays, a stock solution of 1000 µg/mL HAE-*Hbrasileto* was prepared in DMSO (Rotipuran ≥ 99.8%, p.a.). Concentration–response curves were obtained for worms exposed to Cd (50–1000 µM) or HAE-*Hbrasiletto* (50–5000 µg/mL) alone. To evaluate the protective effect of HAE-*Hbrasiletto* on Cd-induced toxicity in *C. elegans*, three groups of treatments were generated: the first corresponded to worms exposed to the HAE-*Hbrasileto* alone, the second consisted of worms exposed to the Cd solution, and a third one was a combination of Cd (50–1000) with 500 or 1000 µg/mL HAE-*Hbrasiletto*. Control vehicles consisted of K-medium and 1% DMSO for Cd and HAE-*Hbrasiletto*, respectively. Three independent bioassays were carried out for each endpoint, and three replicates per concentration were used in each experiment.

### Protective effects on *C. elegans*

The solutions containing the distinct concentrations of HAE-*Hbrasiletto*, Cd or the mixture of both (HAE-*Hbrasileto* + Cd) were deposited in transparent 96-well plates. Required number of *C. elegans* larvae were added to each well. After exposure, lethality, locomotion, growth, reproduction (laid egg number) and DAF-16 nuclear translocation were assessed.

### Lethality

Synchronized *C. elegans* from the L4 larval (N2) stage were exposed for 24 h to the working groups: (1) control vehicle, (2) HAE-*Hbrasiletto* (0–5.000 mg/mL), (3) Cd (50–1.000 µM) or (4) the combination of Cd and the extract, each well containing 12 ± 2 worms. After exposure, the number of live and dead worms was counted. Dead larvae were those that did not move after gentle touch and did not display pharyngeal pumping [26].

### Locomotion (body bends)

Locomotion was assessed by recording the body bend frequency of the nematode as previously described [27]. The age-synchronized N2 worms at the L4 larval stage were exposed to different treatments for 24 h at 20 °C in the absence of food. A total of 30 worms were individually examined per treatment, and the number of body bends (sinusoidal forward movements) in 20 s was scored for each nematode.

### Body length

Approximately 12 ± 2 synchronized worms in the first larval stage (L1) were placed in a 24-well plate containing different treatments (48 h at 20 °C) and *E. coli* OP50 as a source of food. Afterwards, the worms were immobilized by heat, and their body lengths were measured with a stereomicroscope (Nikon SMZ745T) using ImageJ software to capture worm images [26, 27].

### Reproduction

Larval (L4) stage wild-type N2 worms (12 ± 2) were treated in a transparent 96-well plate for 24 h at 20 °C. After exposure, five worms were individually transferred to Petri plates (NMG agar seeded with *E. coli*) every day for 72 h at 20 °C. The number of eggs and larvae present on each plate was counted using a dissecting microscope [28].

### DAF-16 nuclear translocation

Synchronized TJ356 transgenic larvae at the L4 stage (~ 300) were transferred to nonfluorescent black 96-well plates containing different treatments and incubated at 20 °C for 24 h. After the exposure time, the worms were immobilized on slide plates using 10 mM sodium azide. Subcellular localization of DAF-16 was assessed on a Nikon ECLIPSE 80i fluorescence microscope. For each group, 40 nematodes were imaged with a 10X objective and classified according to the occurrence of DAF-16 translocation to the nucleus [13]. The results are presented as the fraction (%) of the different phenotypes observed in each group.

### Radical reduction capacity (DPPH)

The radical scavenging activity of the HAE-*Hbrasiletto* was assessed using the 2,2-diphenyl-1-picrylhydrazyl (DPPH) antioxidant assay Kit (Bioquochem, Asturias, Spain) with minor modifications. Five concentrations of the HAE-*Hbrasiletto* (62.5–1000 µg/mL) were tested. A calibration curve prepared utilizing Trolox, a vitamin A analog, was used as a positive control [29]. Twenty microliters of the samples or standards were reacted with 200 µL of the DPPH solution in the dark at room temperature. The absorbance at 517 nm was then measured using a Varioskan™ LUX multimode microplate reader (Thermo Fisher Scientific, Inc., Waltham, MA, USA). The DPPH scavenging activity was calculated using the formula:

$$\mathrm{DPPH radical scavenging activity }\left(\mathrm{\%}\right)=100-[\left(\frac{\mathrm{Asample}-\mathrm{Ablank}}{\mathrm{Acontrol}}\right)*100]$$

where A_sample_ is the absorbance of the sample, A_blank_ is the absorbance of the blank (ethanol and sample) and A_control_ is the absorbance of the DPPH• + solution without the extract [30]. Two independent experiments were carried out with two replicates each.

### Statistical analysis

All data are reported as the mean ± standard error of the mean. Data were checked for normality and homoscedasticity using Shapiro–Wilk and Bartlett's tests, respectively. Mean comparisons between groups were carried out employing ANOVA, followed by Dunnett's multiple comparisons test. Pearson correlation was carried out to establish concentration–response relationships and associations between endpoints. The graphs were generated using GraphPad Prism 8.0 (GraphPad Prism Software, Inc., San Diego, USA). A *p* value < 0.05 was considered statistically significant.

## Results

### Phytochemical analysis

Chemical analysis of the HAE-*Hbrasiletto* by HPLC-QTOF-MS/MS is shown in Table 1. The secondary metabolites tentatively identified from HAE-*Hbrasileto*, classified according to their molecular skeleton, correspond to pterocarpans (brazilein and hematein), protosappanins (protosappanins A, B and C, 10-O-methylprotosappanin B and protosappanin A dimethyl acetal) and sappanins (7,3’,4’-trihydroxy-3-benzyl-2H-chromene). Chromatograms of HAE-*Hbrasileto* (Fig. S1), fragment ions of MS/MS spectra (Table S1), and compound fragment spectrum results (MS/MS) (Fig. S2) are presented in the Supplementary Material.

**Table 1:**
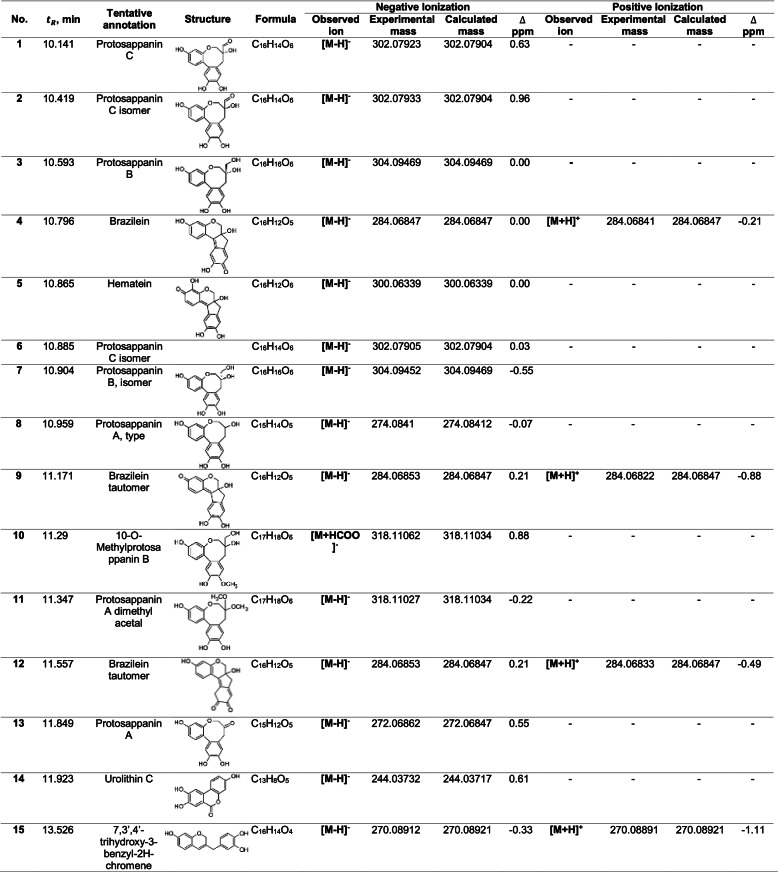
Negative and positive ions of the compounds tentatively identified in HAE-*Hbrasiletto* by HPLC-QTOF-MS/MS

### Protective effects in *C. elegans.*

#### Lethality

**Fig. 1:**
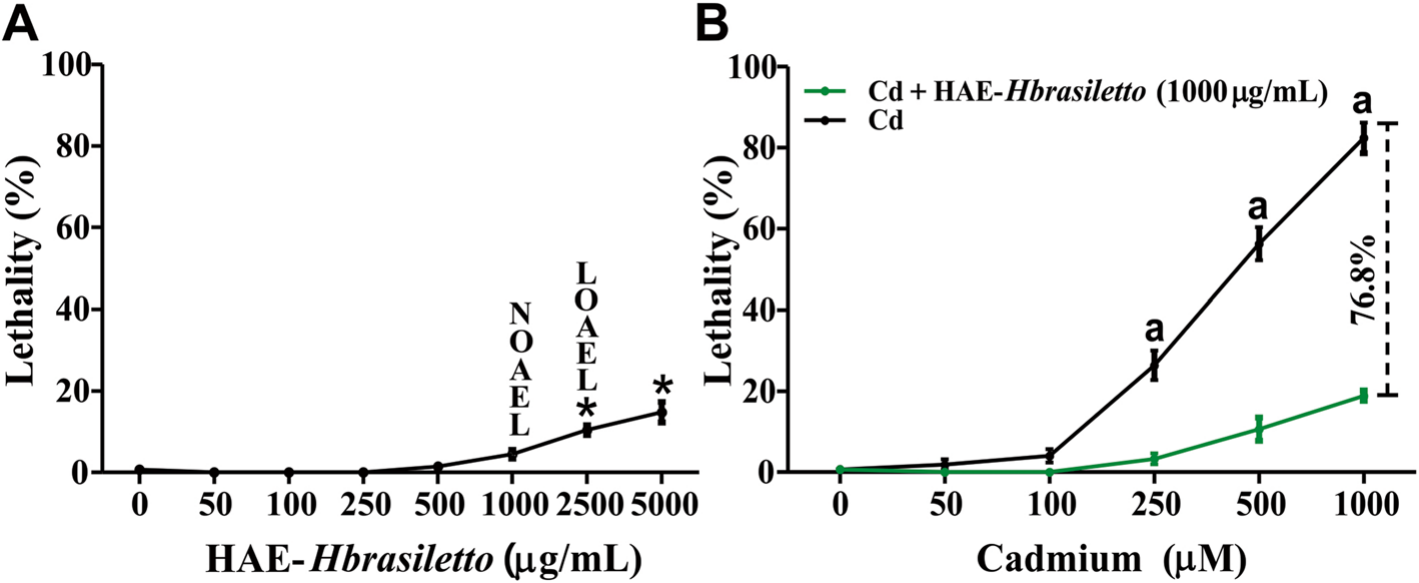
Effect of HAE-*Hbrasiletto* on Cd-induced lethality in *C. elegans.* **A** Lethality in *C. elegans* exposed to HAE-*Hbrasiletto*. **B** HAE-*Hbrasiletto* decreased lethality induced by Cd on *C. elegans*. Significant difference (*p* < 0.05) when compared to vehicle control (*) or Cd treatment (a); NOAEL, no observed adverse effect level; LOAEL, lowest observed adverse effect level

The HAE-*Hbrasiletto* extract induced low lethality in N2 worms (Fig. 1A). The NOAEL was 1000 µg/dL, and the LOAEL was 2500 µg/mL. At the maximum tested concentration (5000 µg/mL), lethality reached 14.7%. The effect of HAE-*Hbrasiletto* on Cd-induced lethality in *C. elegans* is displayed in Fig. 1B. Cadmium alone produced, on average, 81% lethality at the highest evaluated concentration (1000 µm). However, 1000 µg/mL HAE-*Hbrasiletto* significantly reduced the Cd-induced death of the nematodes by 87.5. 79.6 and 76.8% at 250, 500 and 1000 µM Cd, respectively.

#### Physiological endpoints

**Fig. 2:**
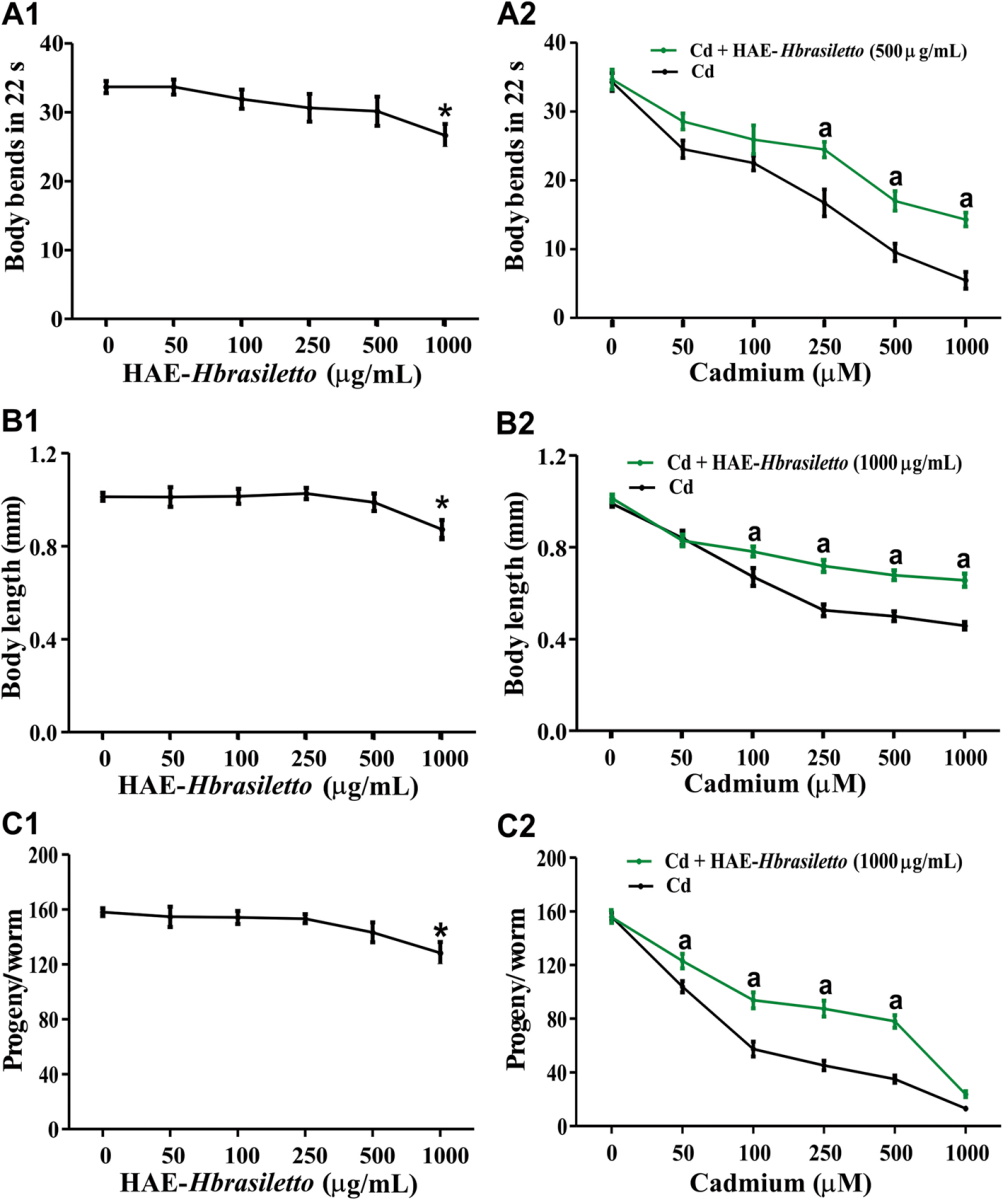
Effects of AE-*Hbrasiletto* on Cd-induced physiological changes in *C. elegans*. **A1**, **B1** and **C1** display the effects of HAE-*Hbrasiletto* alone on locomotion, growth and reproduction. **A2**, **B2** and **C2** show the counteracting effects of HAE-*Hbrasiletto* on Cd-induced physiological changes. Significant difference (*p* < 0.05) when compared to vehicle control (*) or Cd treatment (a)

The effects of HAE-*Hbrasiletto* on key physiological endpoints in *C. elegans* are shown in Fig. 2. Treatment with the HAE-*Hbrasiletto* induced a modest but concentration-dependent effect on worm motility, displaying a significant difference when compared to the control only at 1000 µg/mL (Fig. 2A1). Cadmium has a profound impact on worm locomotion, reducing it up to 84% at 1000 µM Cd. The HAE-*Hbrasiletto*, at a concentration that did not elicit an effect on locomotion (500 µg/mL), was able to recover Cd-induced motility inhibition by 21.8, 21.2 and 25.3%, at 250, 500 and 1000 µM Cd, respectively (Fig. 2A2). The HAE-*Hbrasiletto* did not generate negative effects on nematode growth at concentrations up to 500 µg/mL, and it had little impact (9.7%) at the highest concentration (1000 µg/mL) (Fig. 2B1). Cadmium drastically affected nematode growth, especially at higher concentrations, reducing it up to 53.7% at 1000 µM Cd. The co-treatment extract-Cd recovered the metal-induced decrease in the size of the worms by 9.3, 17.8, 16.4 and 18.4% in 100, 250, 500 and 1000 µM Cd, respectively (Fig. 2B2). The reproductive capacity of *C. elegans* was not altered by HAE-*Hbrasiletto*, except at the highest tested concentration (1000 µg/mL, 8.1%) (Fig. 2C1). The nematodes exposed to Cd drastically decreased the number of progeny (eggs and larva) and presented delays in egg laying even at low concentrations, with decreases ranging from 33.4% to 92.2% at 50–1000 µM (Fig. 2C2). In contrast, in the presence of HAE-*Hbrasiletto*, the Cd-induced reduction in the number of offspring was modestly abrogated, recovering from 17.3 to 28.5% at 50–500 µM Cd. At the highest Cd concentration (1000 µM), the extract did not display recovery capacity.

#### DAF-16 translocation

**Fig. 3:**
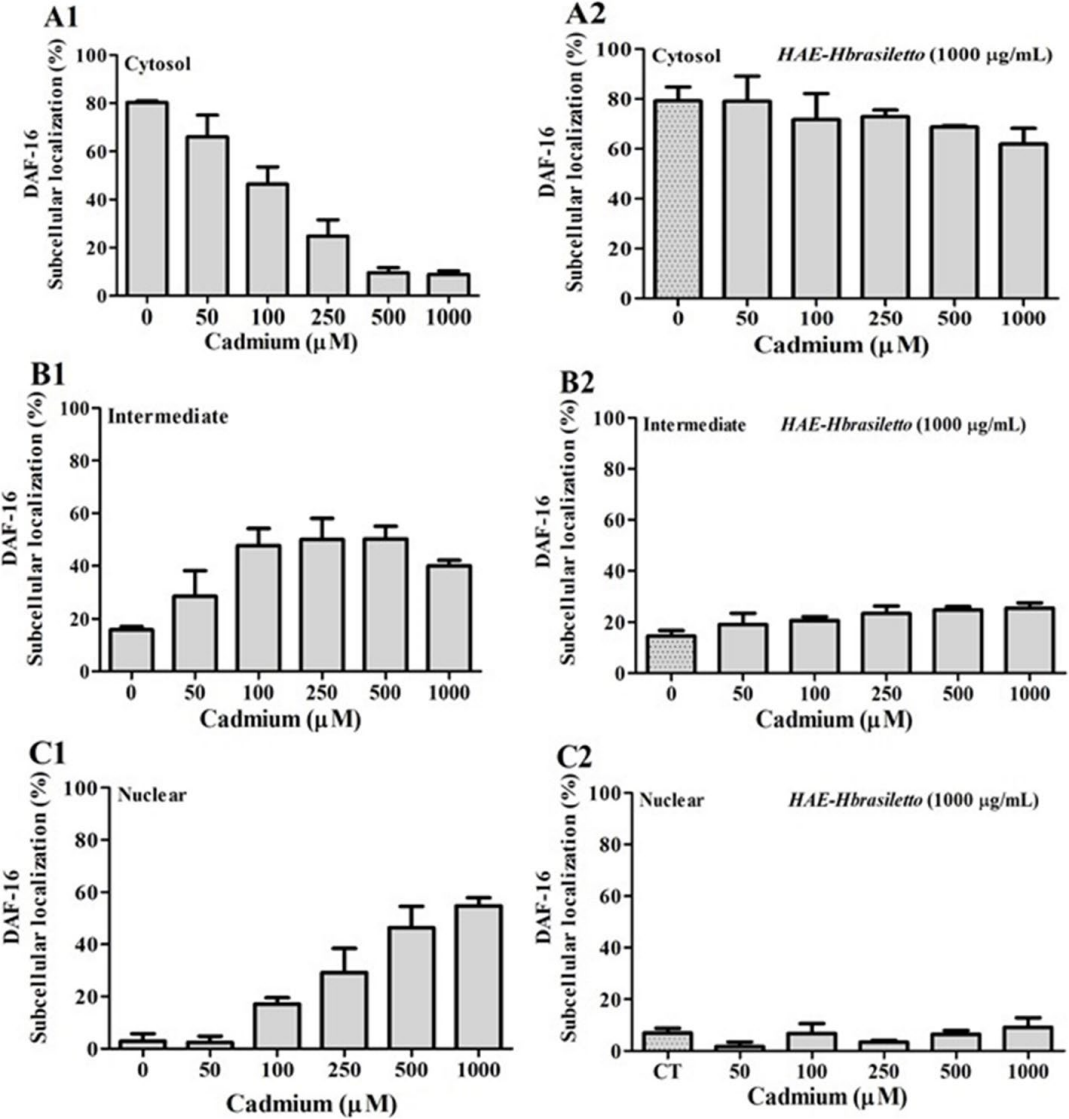
DAF-16 subcellular localization in *C. elegans* under different treatments. Cadmium (1) and the mixture Cd + HAE-*Hbrasiletto* (1000 µg/mL) (2). **A**, **B** and **C** correspond to cytosol, intermediate (cytosol and nuclear) and nuclear DAF-16 localization, respectively

The results of the DAF-16 translocation assay are presented in Fig. 3. Worms exposed to Cd exhibited a concentration-dependent translocation of DAF-16 (Fig. 3A). HAE-*Hbrasiletto* (1000 µg/mL) allowed nematodes to keep most DAF-16 in the cytosol (> 86%), some in the cytosol and the nucleus (intermediate, 13%), and little in the nucleus (1.1%) (Fig. 3B, Column 1, Extract). Cotreatment with Cd and HAE-*Hbrasiletto* halted Cd-induced DAF-16 translocation, avoiding it at Cd concentrations between 50 and 500 µM and decreasing it from 42 to 62% at 500 and 1000 µg/mL Cd, respectively (Fig. 3B)

#### Antioxidant activity assay

**Fig. 4:**
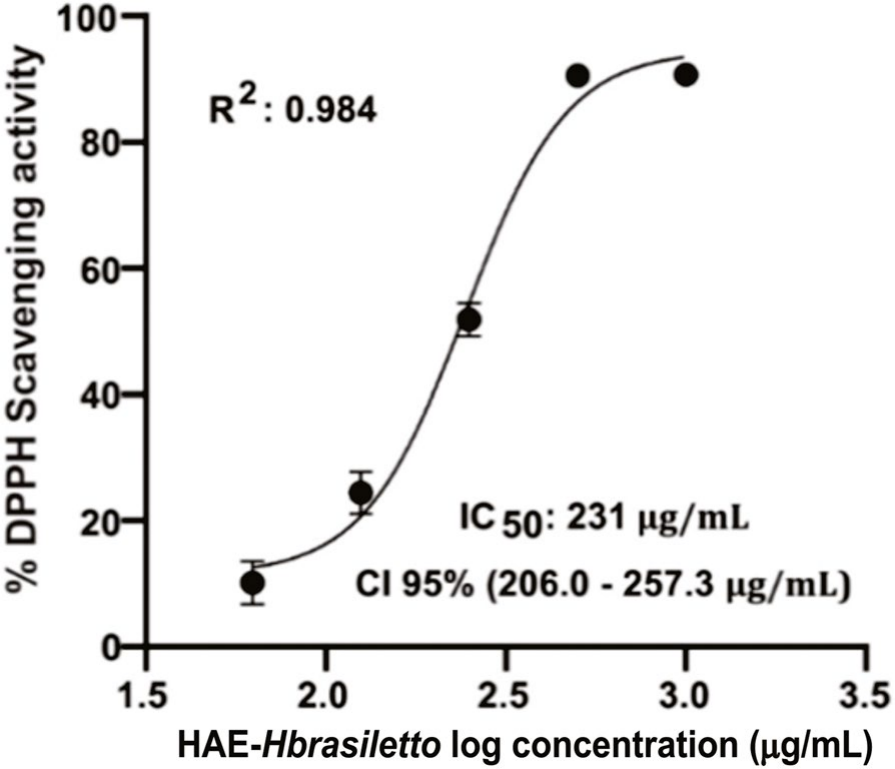
The DPPH radical scavenging activity of HAE-*Hbrasiletto*

The DPPH free radical scavenging activity of HAE-*Hbrasiletto* is shown in Fig. 4. The extract depicted good antioxidant activity, displaying a nonlinear concentration response, with an IC_50_ value of 231 µg/mL (CI 95%: 206–257.3 µg/mL). This chemical activity was lower than that elicited by the water-soluble vitamin E analog Trolox, although it was within the same order of magnitude (IC_50_: 85.3 µg/mL) (Fig. S3).

Correlation analysis of concentration–response data and between different endpoints for Cd exposure are shown in Table S2. Lethality correlated positively with Cd concentration (r = 0.976, *p* = 0.004), and inversely with locomotion (r = -0.947, *p* = 0.015). Moreover, nematode lethality was associated with DAF translocation (r = 0.962, *p* = 0.009).

## Discussion

Despite the recognized place of Colombian flora as one of the most biodiverse in the world, there is a countless number of species, frequently used in traditional medicine, waiting for chemical and pharmacological characterization. This occurs despite the global need for new molecules that could be used to battle disease as well as to counteract toxic effects induced by pollutants. *H. brasiletto* has been suggested to be important for conservation due to its scarcity and medicinal use [31].

HAE-*Hbrasiletto* possesses metabolites that have been reported in several species of the family Fabaceae. Of particular interest are brazilein and protosappanins. The first has a well-known reputation to inhibit inflammation [32] and tumor cell growth [33]. It also has antiviral properties [34], and it has even been proposed as a potential drug candidate against SARS-CoV-2 [35]. Interestingly, brazilein works as a neuroprotector, controlling oxidative stress and promoting myelin regeneration [36]. This may in part explain the blocking effects of HAE-*Hbrasiletto* in Cd-induced locomotion inhibition, as Cd exposure induces structural and chemical changes in myelin [37]. Protosappanin B, an antitumor chemical [38], also exerts neuronal protection by a mechanism involving ubiquitin-dependent p53 protein degradation and mitochondrial homeostasis [39].

Although literature reports have shown that *H. brasiletto* possesses diverse biological properties, such as antiproliferative [19], antiparasitary [23] and antibacterial activities [21], little is known about its role as protective agent against chemical stressors in an in vivo model.

One of the most common elements employed as a chemical stressor in animal models is Cd. In *C. elegans*, Cd has been shown to produce lethality and a reduction in locomotion size and reproduction [40], as also reported here. Several authors have shown that Cd shrinks the serotonergic neuronal body and decreases the expression of tryptophan hydroxylase, a key enzyme in serotonin synthesis, processes that have been recognized as critical for Cd-induced lethality [41]. As Cd-induced locomotion inhibition in the worm is extensively diminished by HAE-*Hbrasiletto*, this mixture has neuroprotective properties.

Body length is highly sensitive to Cd exposure, and HAE-*Hbrasiletto* has a moderate role in counteracting this action. It has been hypothesized that Cd exerts a decrease in nutrient assimilation, leading to a lower growth rate [42]. Interestingly, several authors have observed that the nutrients present in wood ash decrease Cd toxicity [43]. This may suggest that the extract may provide some nutrient-like molecules that could hamper Cd action in the worm.

Reproduction was also improved by HAE-*Hbrasiletto* in *C. elegans* treated with Cd. This metal is known to be a teratogen [44], and in the assayed nematode, fecundity and fertility are frequently damaged by teratogens, chemicals that during early larval development possess slight effects on egg laying and egg quality [45].

An important finding in this work dealt with the inhibitory effect of HAE-*Hbrasiletto* on Cd-induced DAF-16 translocation, which occurs when the worms are subjected to oxidative stress [16]. Cd promotes the production of ROS in different organisms [46]; thus, HAE-*Hbrasiletto* may work as a good antioxidant mixture. Interestingly, the correlation observed between lethality and DAF-16 translocation in Cd-exposed animals suggests that oxidative stress is a key biochemical mechanism controlled by the extract to diminish lethality, a process also supported by its DPPH radical scavenging activity. In fact, major compounds present in the extract, such as brazilein and protosappanins A and B, have been shown to have good antioxidant properties in vitro [47]. These findings add support to the use of natural antioxidants to control ROS production and toxicity induced by Cd [48].

The fact that HAE-*Hbrasiletto* displayed good radical scavenging properties, an excellent capacity to block DAF-16 nuclear translocation, and the ability to ameliorate several Cd-induced physiological changes in *C. elegans*, suggests that it may be a phytotherapeutic alternative in Cd- or metal-induced pathologies mediated by oxidative stress.

## Conclusions

The hydroalcoholic extract of *H. brasiletto* reduced Cd toxicity in *C. elegans* by increasing the survival rate, egg laying and size of worms when exposed to cotreatments. It also displayed good antioxidant activity, which may counteract the ROS-producing activity and Cd-induced toxicity. The beneficial effects of the extract may arise from the presence of well-known antioxidant and neuroprotective molecules, such as brazilein and protosappanin A.

## Supplementary Information

**Additional file 1.** Methods, additional tables, and graphs.

## Data Availability

The supporting data are available from the corresponding author upon reasonable request.

## Abbreviations

CI95%: 95% Confidence interval
DPPH: 1,1-Diphenyl-2-picrylhydrazyl
DMSO: Dimethyl sulfoxide
IC_50_: Half-maximal inhibitory concentration
*HAE-Hbrasiletto*: Hydroalcoholic extract of *Haematoxylum brasiletto*

## References

[1] Lautié E, Russo O, Ducrot P, Boutin JA (2020). Unraveling Plant Natural Chemical Diversity for Drug Discovery Purposes. Front Pharmacol.

[2] Brendler T, Al-Harrasi A, Bauer R (2021). Botanical drugs and supplements affecting the immune response in the time of COVID-19: Implications for research and clinical practice. Phytother Res.

[3] WHO (2021). Traditional Medicine.

[4] Sobeh M, ElHawary E, Peixoto H (2016). Identification of phenolic secondary metabolites from Schotia brachypetala Sond. (Fabaceae) and demonstration of their antioxidant activities in Caenorhabditis elegans. PeerJ.

[5] Adams M, Chammartin M, Hamburger M, Potterat O (2013). Case study of the Swiss flora for prior phytochemical and biological investigations. J Nat Prod.

[6] Noreña-P A, González-Muñoz A, Mosquera-Rendón J, Botero K, Cristancho MA (2018). Colombia, an unknown genetic diversity in the era of big data. BMC Genomics.

[7] Rivera DE, Ocampo YC, Castro JP, Barrios L, Díaz F, Franco LA (2019). A screening of plants used in Colombian traditional medicine revealed the anti-inflammatory potential of *Physalis angulata* calyces. Saudi J Biol Sci.

[8] Ding AJ, Zheng SQ, Huang XB (2017). Current perspective in the discovery of anti-aging agents from natural products. Nat Prod Bioprospect.

[9] Zwirchmayr J, Kirchweger B, Lehner T, Tahir A, Pretsch D, Rollinger JM (2020). A robust and miniaturized screening platform to study natural products affecting metabolism and survival in *Caenorhabditis elegans*. Sci Rep.

[10] Sayed SMA, Siems K, Schmitz-Linneweber C, Luyten W, Saul N (2021). Enhanced healthspan in Caenorhabditis elegans treated with extracts from the traditional chinese medicine plants Cuscuta chinensis Lam. and Eucommia ulmoides Oliv. Front Pharmacol.

[11] Schertzinger G, Zimmermann S, Grabner D, Sures B (2017). Assessment of sublethal endpoints after chronic exposure of the nematode *Caenorhabditis elegans* to palladium, platinum and rhodium. Environ Pollut.

[12] Park HH, Jung Y, Lee SV (2017). Survival assays using *Caenorhabditis elegans*. Mol Cells.

[13] Olivero-Verbel J, De la Parra-Guerra A, Caballero-Gallardo K, Sierra-Marquez L, Fuentes-Lopez K, Franco-Marmolejo J, Jannasch AS, Sepulveda MS, Stashenko E (2021). The aqueous extract of *Fridericia chica* grown in northern Colombia ameliorates toxicity induced by Tergitol on *Caenorhabditis elegans*. Comp Biochem Physiol C Toxicol Pharmacol.

[14] Lagido C, McLaggan D, Flett A, Pettitt J, Glover LA (2009). Rapid sublethal toxicity assessment using bioluminescent *Caenorhabditis elegans*, a novel whole-animal metabolic biosensor. Toxicol Sci.

[15] Jiang Y, Chen J, Wu Y, Wang Q, Li H (2016). Sublethal toxicity endpoints of heavy metals to the nematode *Caenorhabditis elegans*. PLoS ONE.

[16] Kondo M, Yanase S, Ishii T, Hartman PS, Matsumoto K, Ishii N (2005). The p38 signal transduction pathway participates in the oxidative stress-mediated translocation of DAF-16 to *Caenorhabditis elegans* nuclei. Mech Ageing Dev.

[17] De la Parra-Guerra A, Olivero-Verbel J (2020). Toxicity of nonylphenol and nonylphenol ethoxylate on *Caenorhabditis elegans*. Ecotoxicol Environ Saf.

[18] Rivero-Cruz JF (2008). Antimicrobial compounds isolated from *Haematoxylon brasiletto*. J Ethnopharmacol.

[19] Bello-Martínez J, Jiménez-Estrada M, Rosas-Acevedo JL (2017). Antiproliferative activity of *Haematoxylum brasiletto* H. Karst Pharmacogn Mag.

[20] Satarug S, Vesey DA, Gobe GC (2017). Kidney cadmium toxicity, diabetes and high blood pressure: The perfect storm. Tohoku J Exp Med.

[21] Yasunaka K, Abe F, Nagayama A (2005). Antibacterial activity of crude extracts from Mexican medicinal plants and purified coumarins and xanthones. J Ethnopharmacol.

[22] Rosas-Piñón Y, Mejía A, Díaz-Ruiz G, Aguilar MI, Sánchez-Nieto S, Rivero-Cruz JF (2012). Ethnobotanical survey and antibacterial activity of plants used in the altiplane region of Mexico for the treatment of oral cavity infections. J Ethnopharmacol.

[23] Molina-Garza ZJ, Bazaldúa-Rodríguez AF, Quintanilla-Licea R, Galaviz-Silva L (2014). Anti-Trypanosoma cruzi activity of 10 medicinal plants used in northeast Mexico. Acta Trop.

[24] Valko M, Rhodes CJ, Moncol J, Izakovic M, Mazur M (2006). Free radicals, metals and antioxidants in oxidative stress-induced cancer. Chem Biol Interact.

[25] Stuhr NL, Curran SP (2020). Bacterial diets differentially alter lifespan and healthspan trajectories in C. elegans. Commun Biol..

[26] Miao X, Zhang X, Yuan Y (2020). The toxicity assessment of extract of Peganum harmala L. seeds in Caenorhabditis elegans. BMC Complement Med Ther..

[27] Acosta-Coley I, Duran-Izquierdo M, Rodriguez-Cavallo E, Mercado-Camargo J, Mendez-Cuadro D, Olivero-Verbel J (2019). Quantification of microplastics along the Caribbean coastline of Colombia: Pollution profile and biological effects on *Caenorhabditis elegans*. Mar Pollut Bull.

[28] Lee SY, Kang K (2017). Measuring the effect of chemicals on the growth and reproduction of *Caenorhabditis elegans*. J Vis Exp.

[29] Milardović S, Iveković D, Grabarić BS (2006). A novel amperometric method for antioxidant activity determination using DPPH free radical. Bioelectrochemistry.

[30] Zhang Y, Li Q, Xing H (2013). Evaluation of antioxidant activity of ten compounds in different tea samples by means of an on-line HPLC–DPPH assay. Int Food Res J.

[31] Monroy-Ortiz C, García-Moya E, Romero-Manzanares A, Luna-Cavazos M, Monroy R (2018). Traditional and formal ecological knowledge to assess harvesting and conservation of a Mexican tropical dry forest. J Environ Manage.

[32] Kim KJ, Yoon KY, Yoon HS, Oh SR, Lee BY (2015). Brazilein suppresses inflammation through inactivation of IRAK4-NF-κB pathway in LPS-Induced Raw264.7 macrophage cells. Int J Mol Sci..

[33] Mou Z, Wang Y, Li Y (2019). Brazilein induces apoptosis and G1/G0 phase cell cycle arrest by up-regulation of miR-133a in human vestibular schwannoma cells. Exp Mol Pathol.

[34] Liu AL, Shu SH, Qin HL, Lee SM, Wang YT, Du GH. *In vitro* anti-influenza viral activities of constituents from *Caesalpinia sappan*. Planta Med. 2009;75(4):337–9. 10.1055/s-0028-1112208. 10.1055/s-0028-1112208 19148862

[35] Kurniawan E, Krihariyani D (2021). Working goal of Brazilein sappan wood as a candidate for SARS-coV-2 antivirus drug against spike (S) glycoprotein, papain-like proteinase, and main protease: *In silico study*. J Adv Pharm Technol Res.

[36] Jian C, Zhang L, Jinlong L, Bo T, Liu Z (2020). Effects of brazilein on PSD-95 protein expression and neurological recovery in mice after sciatic nerve injury. Neurosci Lett.

[37] Monaco A, Grimaldi MC, Ferrandino I (2016). Neuroglial alterations in the *zebrafish* brain exposed to cadmium chloride. J Appl Toxicol.

[38] Yang X, Zhao L, Zhang T (2019). Protosappanin B promotes apoptosis and causes G1 cell cycle arrest in human bladder cancer cells. Sci Rep.

[39] Zeng KW, Liao LX, Zhao MB (2015). Protosappanin B protects PC12 cells against oxygen-glucose deprivation-induced neuronal death by maintaining mitochondrial homeostasis via induction of ubiquitin-dependent p53 protein degradation. Eur J Pharmacol.

[40] Moyson S, Vissenberg K, Fransen E, Blust R, Husson SJ (2018). Mixture effects of copper, cadmium, and zinc on mortality and behavior of *Caenorhabditis elegans*. Environ Toxicol Chem.

[41] Wang S, Chu Z, Zhang K, Miao G (2018). Cadmium-induced serotonergic neuron and reproduction damages conferred lethality in the nematode *Caenorhabditis elegans*. Chemosphere.

[42] Nørhave NJ, Spurgeon D, Svendsen C, Cedergreen N (2012). How does growth temperature affect cadmium toxicity measured on different life history traits in the soil nematode *Caenorhabditis elegans?*. Environ Toxicol Chem.

[43] Johansen JL, David MF, Ekelund F, Vestergård M (2019). Wood ash decreases cadmium toxicity to the soil nematode *Caenorhabditis elegans*. Ecotoxicol Environ Saf.

[44] Kumar S, Sharma A (2019). Cadmium toxicity: effects on human reproduction and fertility. Rev Environ Health.

[45] Killeen A, Marin de Evsikova C (2016). Effects of sub-lethal teratogen exposure during larval development on egg laying and egg quality in adult *Caenorhabditis elegans*. F1000Res.

[46] Mohajeri M, Rezaee M, Sahebkar A (2017). Cadmium-induced toxicity is rescued by curcumin: A review. BioFactors.

[47] Sasaki Y, Hosokawa T, Nagai M, Nagumo S (2007). *In vitro* study for inhibition of NO production about constituents of *Sappan Lignum*. Biol Pharm Bull.

[48] Unsal V, Dalkıran T, Çiçek M, Kölükçü E (2020). The role of natural antioxidants against reactive oxygen species produced by cadmium toxicity: a review. Adv Pharm Bull.

