# Supplementary material for: Hydroalcoholic extract of Haematoxylum brasiletto protects Caenorhabditis elegans from cadmium-induced toxicity
Source: BMC Complement Med Ther. 2022 Jul 11;22:184. doi: 10.1186/s12906-022-03654-6 (PMC9272861; doi:10.1186/s12906-022-03654-6)
Supplement: Supplementary file 1 — Additional file 1. Methods, additional tables, and graphs. [file 12906_2022_3654_MOESM1_ESM.docx]

**SUPPLEMENTARY MATERIAL**

**Hydroalcoholic extract of *Haematoxylum brasiletto* protects**

***Caenorhabditis elegans* from cadmium-induced toxicity**

Authors

Margareth Duran-Izquierdo^1^, María Taboada-Alquerque^1^, Lucellys Sierra-Marquez^1^,

Neda Alvarez-Ortega^1^, Elena Stashenko^2^, and Jesus Olivero-Verbel^1^

1. Environmental and Computational Chemistry Group, School of Pharmaceutical Sciences, Zaragocilla Campus, University of Cartagena, Cartagena, 130014, Colombia.
2. Center for Chromatography and Mass Spectrometry, CROM-MASS, CIBIMOL-CENIVAM, Industrial University of Santander, Carrera 27, Calle 9, Building 45, Bucaramanga 680002, Colombia.

Corresponding Author:

Prof. Jesus Olivero-Verbel. Ph.D.

Environmental and Computational Chemistry Group

School of Pharmaceutical Sciences

Zaragocilla Campus

University of Cartagena

Cartagena, 130015.

Colombia

E-mail: joliverov@unicartagena.edu.co

Phone: 57-3185359815

Submitted to: **BMC Complementary Medicine and Therapies**.

**METHODS**

1. **Analysis of the HAE-*Hbrasiletto* by HPLC-QTOF-MS/MS**

The freeze-dried HAE-*Hbrasiletto* (6 mg) was diluted in 3 mL of a mixture of water and acetonitrile 50:50. Diluted sample was shaken until homogenization, followed by centrifugation at 16.000×g for 8 min, the supernatant was filtered through a 0.20 μm pore size membrane and transferred to an autosampler vial. The extract was separated and analyzed using a 1260 Infinity HPLC (Agilent Technologies), coupled to a 6530 quadrupole time-of-flight (q-TOF) mass spectrometer detector (Agilent Technologies), with Electrospray Ionization (ESI), operated in positive and negative ion modes. Separation was carried out using an Infinity Lab Poroshell 120 EC-C18 column, 4.6 x 100 mm, 2.7 μm particle size (Agilent technologies, USA). The column temperature was maintained at 40 °C. The mobile phase consisted of water with 0.1% formic acid (A) and acetonitrile with 0.1% formic acid (B). Analysis started with 95:5 A: B, held for 1 min, followed by changing linearly up to 5:95 in 12 min, then returned to 95:5 A: B in 3 min, and held until 21 min. Flow was 0.3 mL/min and the injection volume 5 μL. The conditions for the mass detector were as follows: Capillary voltage +3.5 kV, nitrogen gas temperature 320 °C, drying gas flow rate 8.0 L/min, nebulizer gas pressure 35 psig, fragmentor voltage 135 V, skimmer 65 V, and OCT RF 750 V. MS/MS Data Acquisition mode was used to assist compound identification. Mass range in MS and MS/MS experiments were set at m/z 100-1200 and 50-1200 at 3 spectra/s, respectively. MS and MS/MS data were collected using Agilent MassHunter Acquisition software (version 10.1). The data obtained was processed with the Agilent MassHunter Qualitative Analysis 10.0. Peak annotations were performed using the METLIN (metlin.scripps.edu) metabolite databases with a mass error of less than 5 ppm. Compound identification was based on the exact masses and MS/MS spectra of the target compounds [1;6]


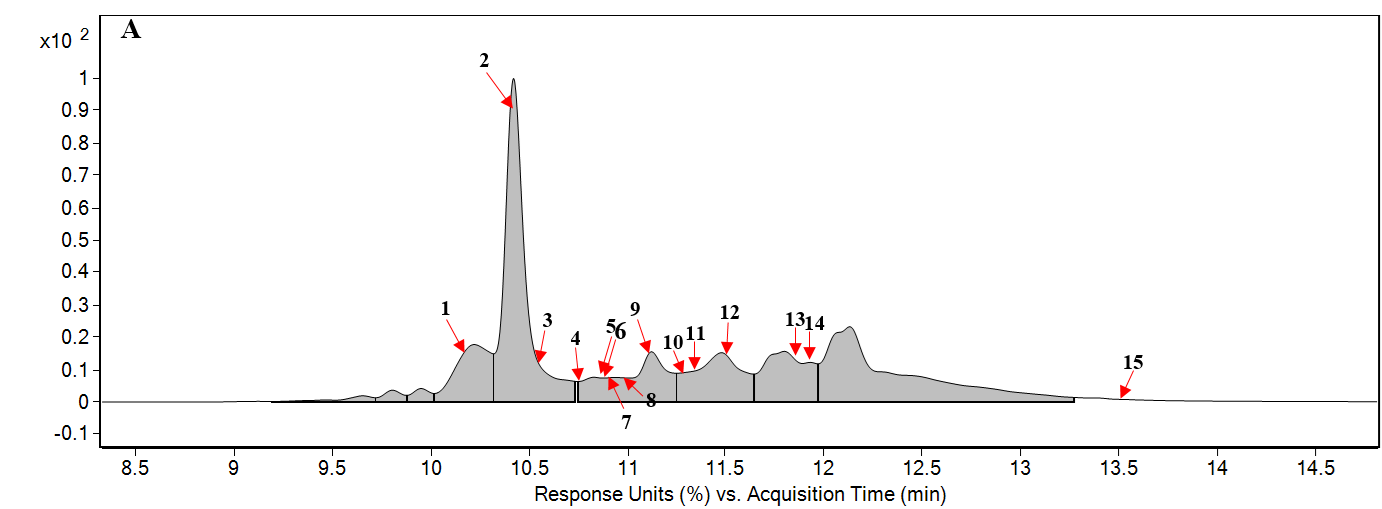


**
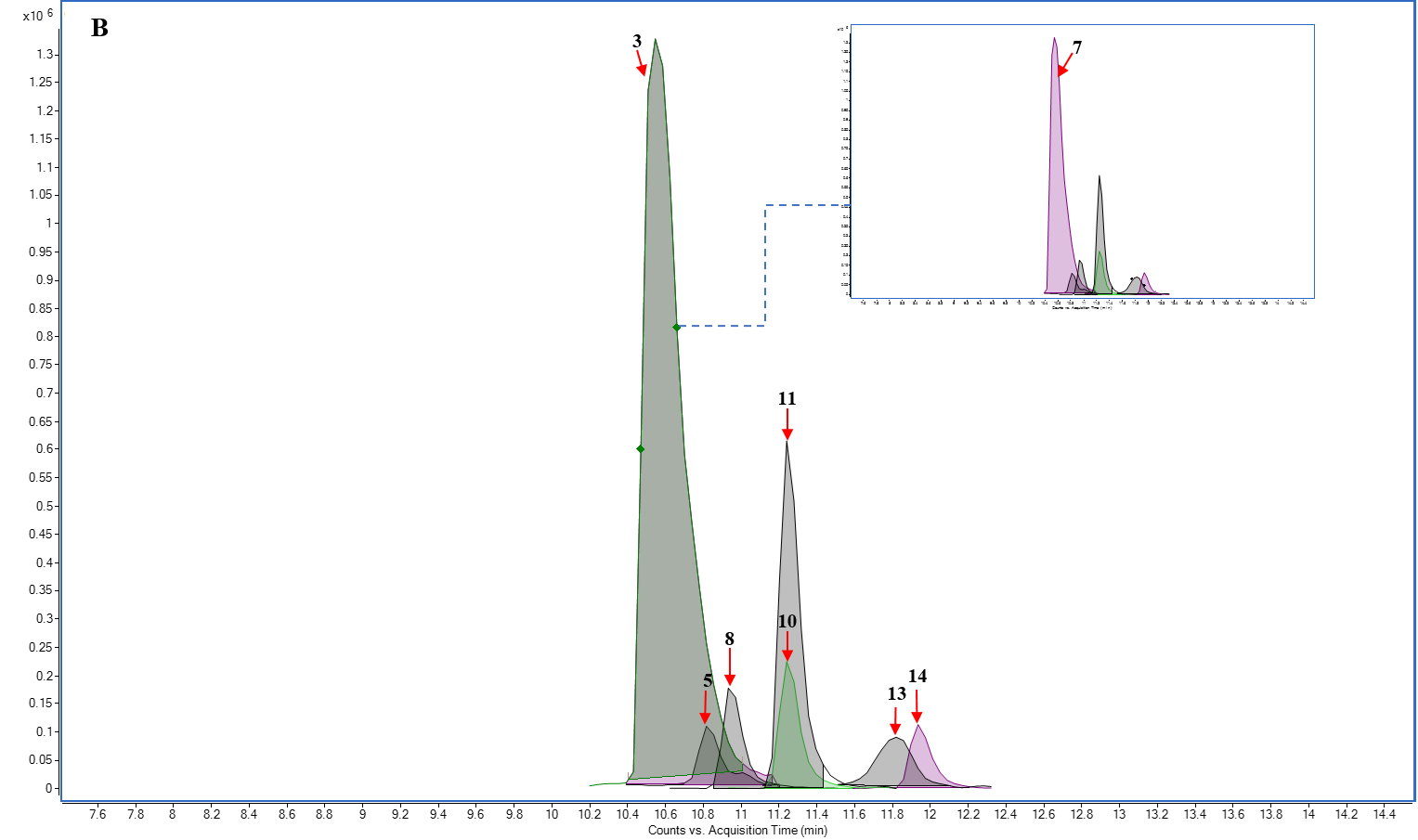
**

**Figure S1.** Chromatograms of Haematoxylum Brasiletto. **A.** Chromatogram from variable wavelength UV-vis detectors operated at 280 nm. **B.** Extract chromatogram Rt: 10-12.5 min. (extracted ion chromatogram, EIC), obtained by HPLC-ESI-QTOF in negative ion mode.

**Table S1.** Fragment ions of chemical constituents tentatively identified in the HAE-Hbrasiletto by HPLC-QTOF-MS/MS.

| No. Fig.  S5B | Fragment ions | | | Identification  criteria | References |
| --- | --- | --- | --- | --- | --- |
|  | **Fragment type** | **Fragment**  **formula** | **m/z** |  |  |
| 1 | [M-H]^-^  [M-H-H_2_O]^-^  [M-H-C_3_H_4_O_2_]^-^  [M-H-C_2_H_4_O_2_]^-^ | [C_16_H_13_O_6_]^-^  [C_16_H_11_O_5_]^-^  [C_13_H_9_O_4_]^-^  [C_14_H_9_O_4_]^-^ | 301.0723  283.0612  229.0503  241.05 | a | Nagai, Nagumo, 1987; Lech and Fornal, 2020. |
| 2 | [M-H]^-^  [M-H-H_2_O]^-^  [M-H-C_3_H_4_O_2_]^-^  [M-H-C_2_H_4_O_2_]^-^ | [C_16_H_13_O_6_]^-^  [C_16_H_11_O_5_]^-^  [C_13_H_9_O_4_]^-^  [C_14_H_9_O_4_]^-^ | 301.0723  283.0612  229.0503  241.05 | a | Nagai, Nagumo, 1987; Lech and Fornal, 2020. |
| 3 | [M-H]^-^  [M-H-C_2_H_4_O_2_]^-^  [M-H-C_3_H_4_O_2_]^-^ | [C_16_H_15_O_6_]^-^  [C_14_H_11_O_4_]^-^  [C_13_H_11_O_4_]^-^ | 303.0875  243.0658  231.0558 | b | Deng et al., 2013. |
| 4 | [M-H]^-^  [M-H-H_2_O]^-^  [M-H-CO]^-^  [M-H-C_6_H_6_O_2_]^-^ | [C_16_H_11_O_5_]^-^  [C_16_H_9_O_4_]^-^  [C_15_H_11_O_4_]^-^  [C_10_H_5_O_3_]^-^ | 283.0615  265.0504  255.0645  173.0223 | b | Lech and Fornal, 2020. |
| 5 | [M-H]^-^  [M-H-H_2_O]^-^  [M-H-CO]^-^  [M-H-C_10_H_6_O_4_]^-^ | [C_16_H_11_O_6_]^-^  [C_16_H_9_O_5_]^-^  [C_15_H_11_O_5_]^-^  [C_6_H_5_O_2_]^-^ | 299.0566  281.0447  271.0584  109.0286 | b | Hulme et al., 2005; Rosenberg, 2008. |
| 6 | [M-H]^-^  [M-H-H_2_O]^-^  [M-H-C_3_H_4_O_2_]^-^ | [C_16_H_13_O_6_]^-^  [C_16_H_11_O_5_]^-^  [C_13_H_9_O_4_]^-^ | 301.0611  283.0608  229.0502 |  | Nagai, Nagumo, 1987; Lech and Fornal, 2020. |
| 7 | [M-H]^-^  [M-H-C_2_H_4_O_2_]^-^  [M-H-C_3_H_4_O_2_]^-^ | [C_16_H_15_O_6_]^-^  [C_14_H_11_O_4_]^-^  [C_13_H_11_O_4_]^-^ | 303.0875  243.0658  231.0558 | b | Deng et al., 2013; Zhang et al., 2017. |
| 8 | [M-H]^-^  [M-H-C_2_H_3_O]^-^  [M-H-C_2_H_4_O]^-^ | [C_15_H_13_O_5_]^-^  [C_13_H_10_O_4_]^-^  [C_13_H_9_O_4_]^-^ | 273.0773  230.0577  229.0500 | a | Tamburini, 2019. |
| 9 | [M-H]^-^  [M-H-H_2_O]^-^  [M-H-CO]^-^  [M-H-C_6_H_6_O_2_]^-^ | [C_16_H_11_O_5_]^-^  [C_16_H_9_O_4_]^-^  [C_15_H_11_O_4_]^-^  [C_10_H_5_O_3_]^-^ | 283.0615  265.0504  255.0645  173.0223 | b | Lech and Fornal, 2020. |
| 10 | [M+HCOO]^-^  [M-H]^-^  [M-H-CH_3_]^-^  [M-H-C_4_H_8_O_2_]^-^ | [C_18_H_19_O_8_]^-^  [C_17_H_17_O_6_]^-^  [C_16_H_14_O_6_]^-^  [C_13_H_9_O_4_]^-^ | 363.1095  317.1026  302.0794  229.0501 | a |  |
| 11 | [M-H]^-^  [M-H-CH_3_]^-^  [M-H-CH_5_O]^-^  [M-H-C_4_H_8_O_2_]^-^ | [C_17_H_17_O_6_]^-^  [C_16_H_14_O_6_]^-^  [C_16_H_12_O_5_]^-^  [C_13_H_9_O_4_]^-^ | 317.1026  302.0794  284.0679  229.0501 | a |  |
| 12 | [M-H]^-^  [M-H-H_2_O]^-^  [M-H-CO]^-^  [M-H-C_6_H_6_O_2_]^-^ | [C_16_H_11_O_5_]^-^  [C_16_H_9_O_4_]^-^  [C_15_H_11_O_4_]^-^  [C_10_H_5_O_3_]^-^ | 283.0615  265.0504  255.0645  173.0223 | b | Lech and Fornal, 2020. |
| 13 | [M-H]^-^  [M-H-H_2_O]^-^  [M-H-CH_2_CO]^-^  [M-H-C_3_H_2_O_2_]^-^ | [C_15_H_11_O_5_]^-^  [C_15_H_9_O_4_]^-^  [C_13_H_9_O_4_]^-^  [C_12_H_9_O_3_]^-^ | 271.0616  253.0486  229.0498  201.0545 | b | Tamburini, 2019. |
| 14 | [M-H]^-^  [M-H-CO]^-^  [M-H-CO_2_]^-^ | [C_13_H_7_O_5_]^-^  [C_12_H_7_O_4_]^-^  [C_12_H_7_O_3_]^-^ | 243.0303  215.0330  199.0394 | b | Tamburini, 2019.  Lech & Fornal, 2020. |
| 15 | [M-H]^-^  [M-H-C_6_H_6_O_2_]^-^  [M-H-C_10_H_8_O_2_]^-^  [M-H-C_7_H_6_O_2_]^-^ | [C_16_H_13_O_4_]^-^  [C_10_H_7_O_2_]^-^  [C_6_H_5_O_2_]^-^  [C_9_H_7_O_2_]^-^ | 269.0819  159.0443  109.0294  147.0448 | b | Tamburini, 2019. |

^a^ Tentative identification based on m/z negative mode, reported in the literature for *H. brasiletto* or other species of the *Fabaceae* family, such as *Caesalpinia sappan*. ^b^ Tentative identification based on the fragmentation pattern study (ESI-QTOF) and data reported in scientific articles.

**Figure S2.** Compound fragment spectrum results (MS/MS)

| **Compound 1**  Retention time: 10.141  Monoisotopic mass: 302.0792  Formula: C_16_H_14_O_6_  Tentative annotation: Protosappanin C | 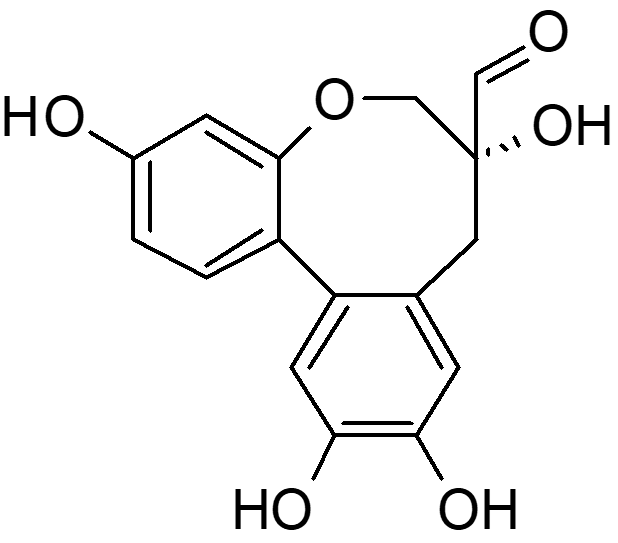 |
| --- | --- |

| **Compound 2**  Retention time: 10.419  Monoisotopic mass: 302.0792  Formula: C_16_H_14_O_6_  Tentative annotation: Protosappanin C isomer | 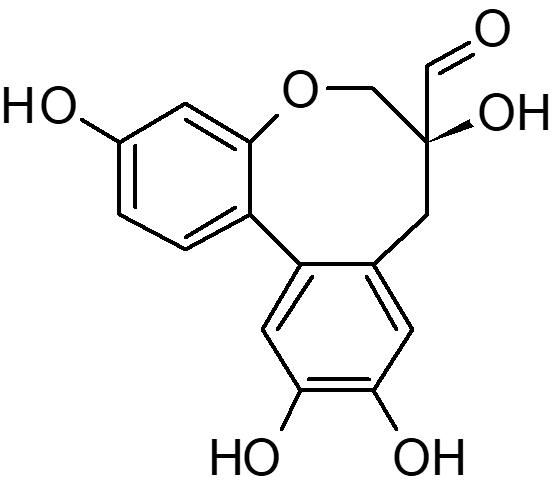 |
| --- | --- |

| **Compound 3**  Retention time: 10.586  Monoisotopic mass: 304.0947  Formula: C_16_H_16_O_6_  Tentative annotation: Protosappanin B | 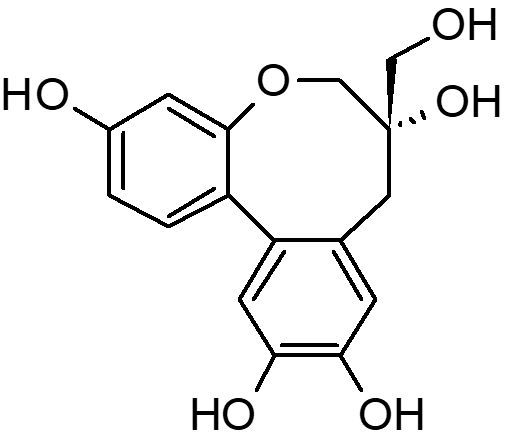 |
| --- | --- |

| **Compound 4**  Retention time: 10.651  Monoisotopic mass: 284.0683  Formula: C_16_H_12_O_5_  Tentative annotation: Brazilein | 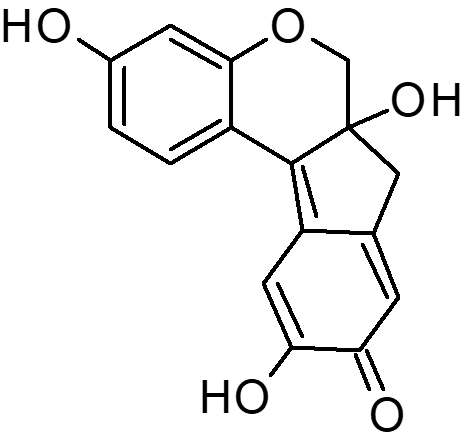 |
| --- | --- |

| **Compound 5**  Retention time: 10.8605  Monoisotopic mass: 300.0634  Formula: C_16_H_12_O_6_  Tentative annotation: Hematein | 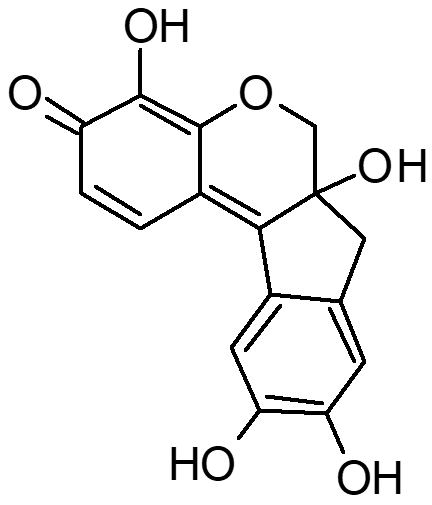 |
| --- | --- |

| **Compound 6**  Retention time: 10.8605  Monoisotopic mass: 302.0791  Formula: C_16_H_14_O_6_  Tentative annotation: Protosappanin C isomer |  |
| --- | --- |

| **Compound 7**  Retention time: 10.889  Monoisotopic mass: 304.0945  Formula: C_16_H_16_O_6_  Tentative annotation: Protosappanin B isomer | 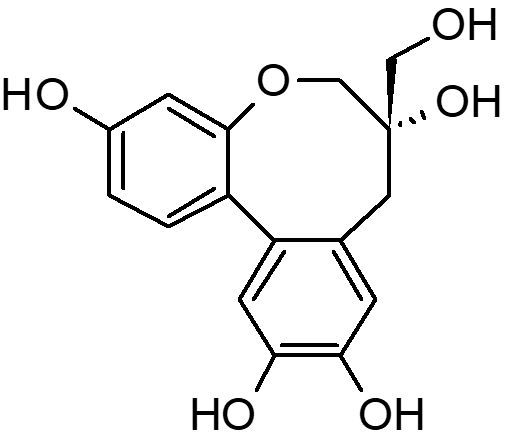 |
| --- | --- |

| **Compound 8**  Retention time: 10.964  Monoisotopic mass: 274.0841  Formula: C_15_H_14_O_5_  Tentative annotation: Protosappanin A-type | 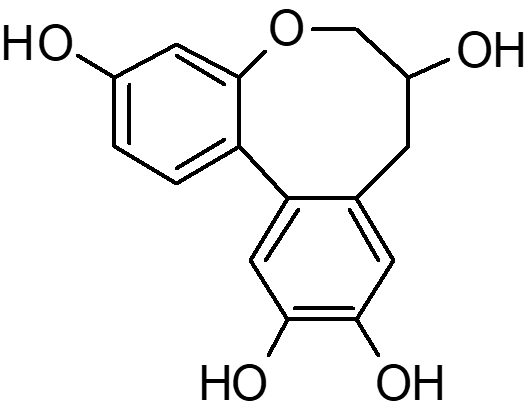 |
| --- | --- |

| **Compound 9**  Retention time: 11.224  Monoisotopic mass: 284.0685  Formula: C_16_H_12_O_5_  Tentative annotation: Brazilein tautomer | 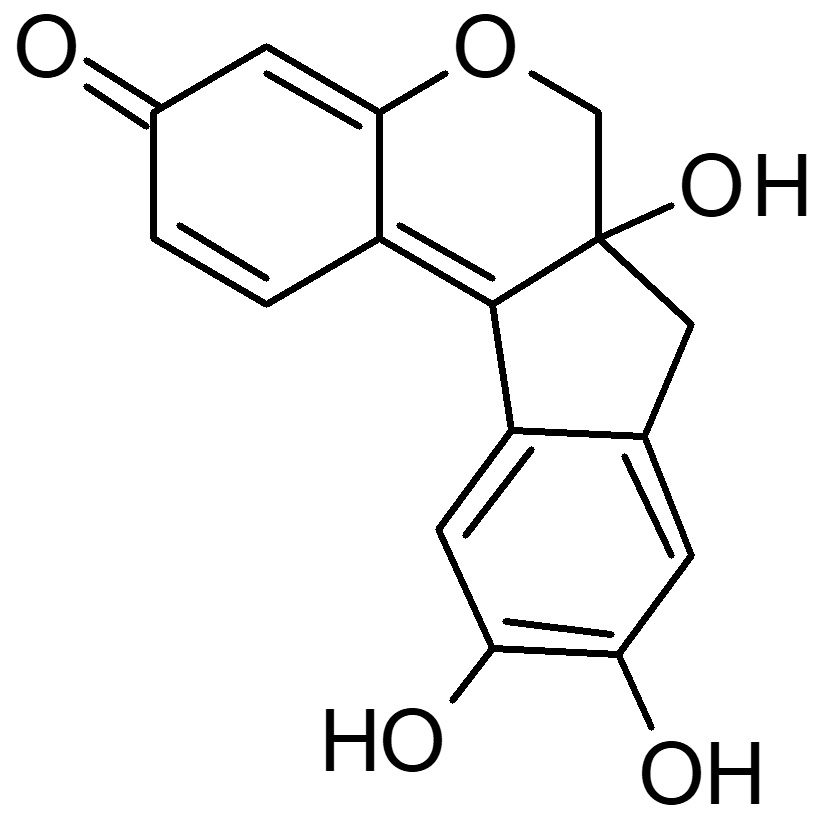 |
| --- | --- |

| **Compound 10**  Retention time: 11.295  Monoisotopic mass: 318.1106  Formula: C_17_H_18_O_6_  Tentative annotation: 10-O-methylprotosappanin B | 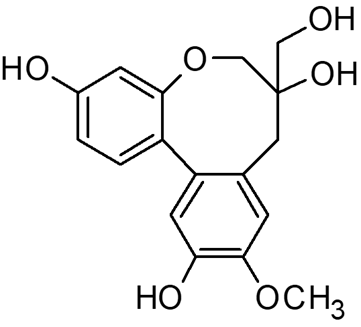 |
| --- | --- |

| **Compound 11**  Retention time: 11.347  Monoisotopic mass: 318.1106  Formula: C_17_H_18_O_6_  Tentative annotation: | 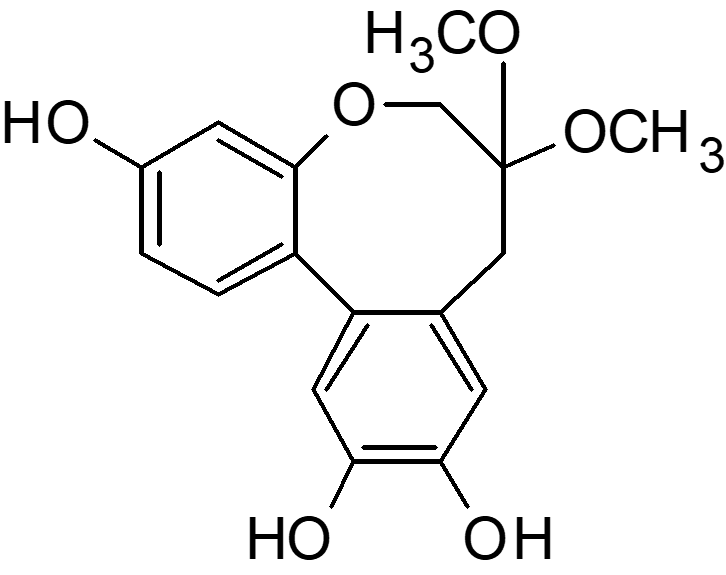 |
| --- | --- |

| **Compound 12**  Retention time: 11.610  Monoisotopic mass: 284.0685  Formula: C_16_H_12_O_5_  Tentative annotation: Brazilein tautomer | 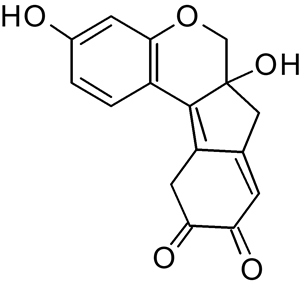 |
| --- | --- |

| **Compound 13**  Retention time: 11.848  Monoisotopic mass: 272.0686  Formula: C_15_H_12_O_5_  Tentative annotation: Protosappanin A | 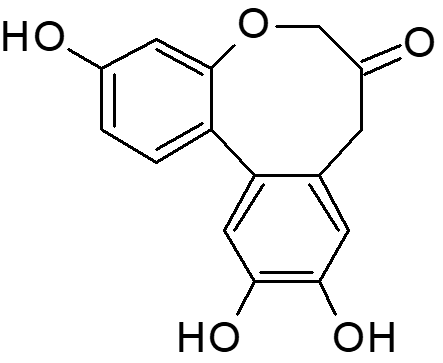 |
| --- | --- |

| **Compound 14**  Retention time: 11.920  Monoisotopic mass: 244.0373  Formula: C_13_H_18_O_5_  Tentative annotation: Urolithin | 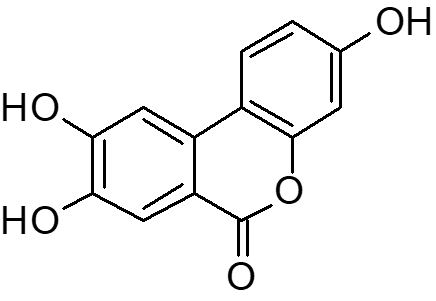 |
| --- | --- |

| **Compound 15**  Retention time: 13.526  Monoisotopic mass: 270.0891  Formula: C_16_H_14_O_4_  Tentative annotation: 7,3,4’-trihydroxy-3-benzyl-2H-chromene | 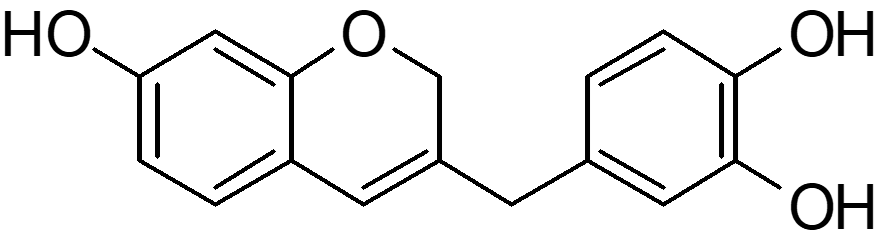 |
| --- | --- |

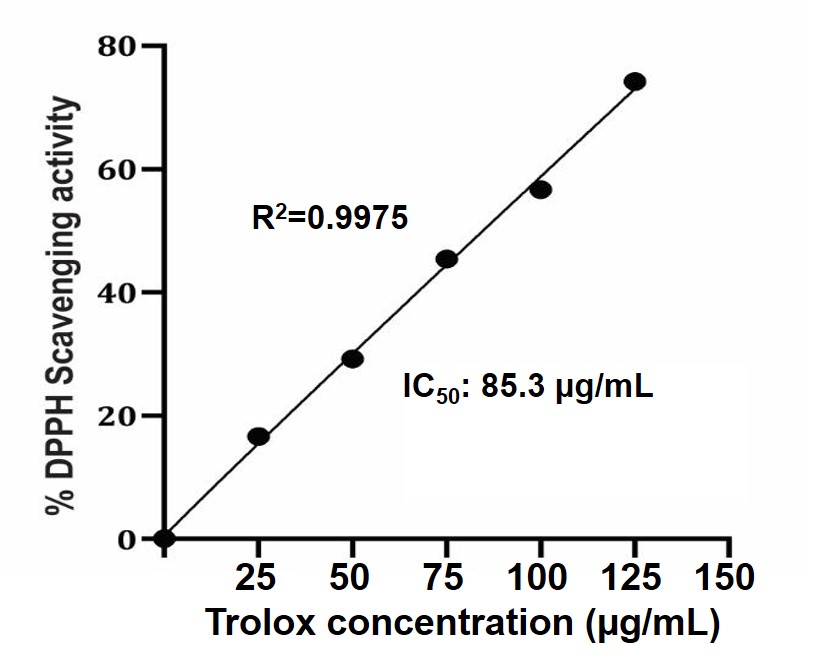


**Figure S3**. The DPPH radical scavenging activity depicted by the ethanolic extract of Trolox standard.

**Table S2.** Pearson correlation obtained for physiological variables vs. Cd concentration and DAF-16 nuclear translocation.

| **Physiological variables of *C. elegans*** | **[Cd]** | **DAF-16**  **Nuclear translocation** |
| --- | --- | --- |
| Lethality | 0.976 (0.004)* | 0.962 (0.009) |
| Locomotion (Body bends) | -0.947 (0.015) | -0.924 (0.025) |
| Body length | -0.778 (0.121) | -0.613 (0.272) |
| Reproduction | -0.837 (0.077) | -0.761 (0.135) |

*. *p*-value in parenthesis.

**References**

**[1]** Deng Z, Wang X, Zhao H, Cui S, Yao Q, Bai H. A validated LC-MS/MS method for rapid determination of brazilin in rat plasma and its application to a pharmacokinetic study. Biomed Chromatogr. 2013;27(6):802-806.

**[2]** Hulme AN, McNab H, Peggie DA, Quye A. Negative ion electrospray mass spectrometry of neoflavonoids. Phytochemistry. 2005;66(23):2766-2770.

**[3]** Lech K, Fornal E. A mass spectrometry-based approach for characterization of red, blue, and purple natural dyes. Molecules*.* 2020;25(14):3223.

**[4]** Nagai M, Nagumo S. Protosappanin C from *Sappan lignum* and absolute configuration of protosappanins. Chem Pharm Bull.1987;35(7):3002-3005.

**[5]** Rosenberg E. Characterisation of historical organic dyestuffs by liquid chromatography–mass spectrometry. Anal Bioanal Chem. 2008;391(1):33-57.

**[6]** Tamburini D. Investigating Asian colourants in Chinese textiles from Dunhuang (7th-10th century AD) by high performance liquid chromatography tandem mass spectrometry–Towards the creation of a mass spectra database. Dyes and Pigments*.* 2019;163:454-474.
